# Small bait traps as accurate predictors of dipteran early colonizers in forensic studies

**DOI:** 10.1093/jis/14.1.77

**Published:** 2014-01-01

**Authors:** Ana Farinha, Catarina G. Dourado, Neiva Centeio, Ana Rita Oliveira, Deodália Dias, Maria Teresa Rebelo

**Affiliations:** 1 University of Lisbon, Faculty of Sciences, 1749-016 Lisbon, Portugal; 2 CESAM - Centre for Environmental and Marine Studies, Lisbon, Portugal

**Keywords:** bait attraction, cytochrome *c* oxidase I, DNA barcoding, forensic entomology

## Abstract

Insect carrion communities vary among habitats and over time. Concerning the dipteran early colonizers of carrion, the use of small bait traps should be accurate because the odors emitted from meat baits should contain many of the volatile organic compounds emitted from the freshly dead mammals. In addition, this kind of trap is easy to replicate and set in position in a given habitat. In the present study, small bait preferences of early Diptera carrion colonizers were examined in an urban biotope. Specifically, three baits were compared (pork muscle, pork liver, and fish flavored cat food) in respect to the number of specimens and species captured and the presence or absence of oviposition at high and low environmental temperatures. A total of 2371 specimens were trapped, primarily belonging to three insect orders, Diptera, Coleoptera, and Hymenoptera. Diptera was the predominant order, with blowflies (Calliphoridae) being the most representative family, followed by filth flies (Muscidae). The pork muscle bait was responsible for the highest number of captures and the highest diversity. The community of Diptera collected with the most efficient bait, pork muscle, was compared with the carrion communities reported in the literature from the Iberian Peninsula. Similar taxonomic species composition was found regarding Calliphoridae species. A specimen from all species morphologically identified were also identified at a molecular level using the cytochrome *c* oxidase I (COI) barcode region, and the sequences were submitted to online databases.

## Introduction

Flies are typically the first insects to colonize decomposing remains. Several studies have indicated that the primary important carrion species belong to a relatively small number of families, namely, Calliphoridae, Muscidae, Sarcophagidae, and Fanniidae ( Amendt et al. 2004 ; Eberhardt and Elliot 2008 ; Battán Horenstein and Salvo 2012 ), but it is important to notice that carrion fly communities vary both geographically and seasonally (Arnaldos et al. 2001; Campobasso et al. 2001 ; Amendt et al. 2004 ; Hwang and Turner 2005 ; Anton et al. 2011 ; Brundage et al. 2011 ). The validation of reference data collected in different locations, even within the same geographic area, has received some attention because variation between habitats, particularly temperature and vegetation, is known to influence insect succession patterns and alter decomposition rates ( Mann et al. 1990 ; Turchetto and Vanin 2004 ; Hwang and Turner 2005 ; Bonacci et al. 2009 ; Brundage et al. 2011 ). Therefore, the knowledge of the primary sarcosaprophagous communities, especially in terms of species composition and relative abundance, of a specific area, can be very useful in medico-legal investigations and veterinary surveillance.

In order to capture forensically important flies, different types of baits and traps have been developed and tested ( Tantawi et al. 1996 ; Arnaldos et al. 2001 ; Oliva 2001 ; Leccese 2004 ; Hwang and Turner 2005 ; Baz et al. 2007 ; d’Almeida and Fraga 2007 ; Matuszewski et al. 2010 ; Brundage et al. 2011 ). Decomposed animal tissues, used as baits, constitute cheap substrates with no ethical implications and have been used in other studies for the collection of early colonizers ( Leccese 2004 ; Hwang and Turner 2005 ; Gruner et al. 2007 ; Brundage et al. 2011 ; George et al 2013 ).

An accurate identification of specimens is the first essential step in forensic studies. Morphologic methods are usually used ( García-Rojo 2004 ; Hwang and Turner 2005 ; Baz et al. 2007 ; Matuszewski et al. 2010 ; Anton et al. 2011 ; Prado e Castro et al. 2011 , 2012), but these methodologies require available identification keys, specialized taxonomic knowledge, and are time consuming. Thus, molecular techniques can be of great help in identifying specimens. The mitochondrial cytochrome *c* oxidase subunit I (COI) gene is well established for this purpose, with reference sequences available for a large number of species ( Amendt et al. 2011 ). Concerning Portugal, only a few molecular studies involving the abundant Diptera species with forensic interest have been performed ( Cainé et al. 2006 , 2009; Ferreira et al. 2011 ; Oliveira et al. 2011 ; Rolo et al. 2013 ). Thus, there is still a lack of COI barcode sequences available in public databases for some groups, namely several families of Diptera.

The present study aims to (1) determine species composition and relative abundance of early carrion colonizers in an urban environment using three different attractants, (2) investigate the effectiveness of the type of trap and bait used in estimating first carrion colonizers, comparing the species obtained from these experiments with those reported in the literature, and (3) identify species through DNA barcode region and add new sequence records to online databases.

## Materials and Methods

### Study area

This study was conducted in Lisbon, Portugal, at the campus of the Faculty of Sciences of the University of Lisbon in a small patch of grass with some ornamental shrubs (38º45’22”N 9º09’30”W).

### Trapping, preservation, and identification

Because insects are cold blooded (poikilothermic), activity and behavior are highly dependent on daily air temperature ( Byrd and Castner 2010 ). Given the Portuguese climate profile—Mediterranean climate, with warm seasons and cold seasons well defined and interspersed with mild seasons—field work experiments were carried out in two groups: the first one with an average daily air temperature above 16ºC (high temperature group; 29th March – 1st April 2011 and 3rd – 6th August 2011), and a second one with an average daily temperature below 16ºC (low temperature group; 13th – 16th December 2011 and 6 ^th^ – 9th January 2012). This threshold was determined based on the meteorological data of daily temperatures in Lisbon in the previous five years. It corresponds to the average daily air temperature of the months with mild temperatures (official data from The Geophysical Institute D. Luís, http://idl.ul.pt/ ). The mean atmospheric temperature measured during the four experiments is presented in Table 1 .

**Table 1.**
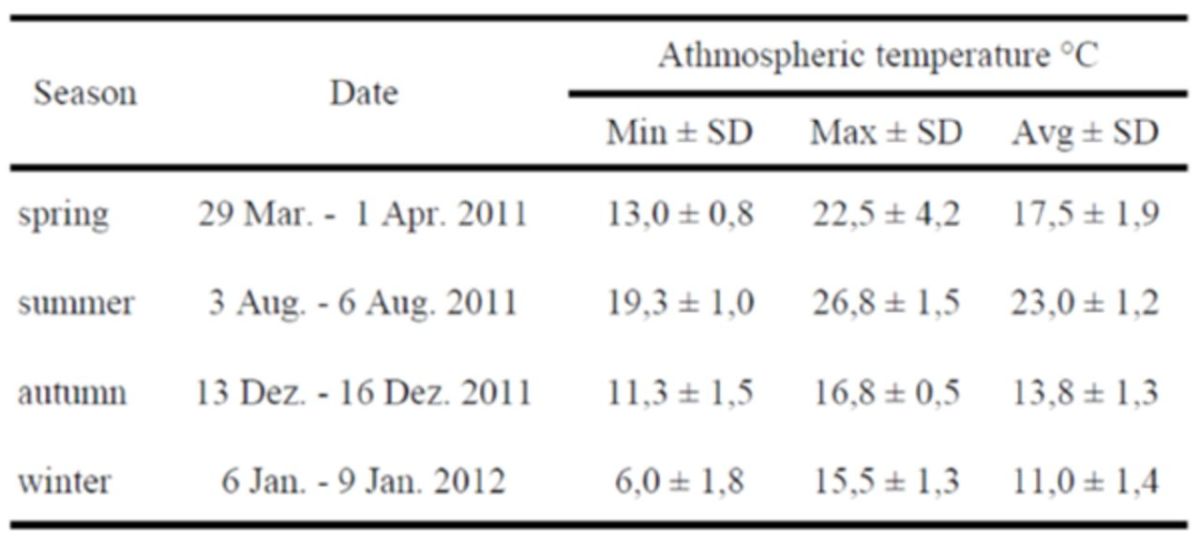
Temperature data (daily average) recorded during the experimental periods (Min - minimum, Max - maximum, Avg - average, SD - standard deviation).

To collect sarcosaprophagous insects, a modified version of the bottle trap was used ( Hwang and Turner 2005 ). Traps consisted of an empty water carboy with a small frontal opening for the entry of the flies. A grid platform was set over the opening to support the bait. The bottom of the carboy was filled with water and some drops of detergent to lower the surface tension of the water and catch the flies that fell down. A transparent plastic tube with a glass vial at the end was inserted into the bottleneck in order to catch adult specimens that flew upwards.

Sixty-four collections were made. In each experiment, four identical traps were set. Three traps were baited, each with 70 g of pork muscle, pork liver or commercial cat food (fish flavor). A fourth trap, unbaited, was used as a control. Each trap was set up to catch flies for 24 hours. After the 24-hour period, traps were closed and insects were collected for identification. The traps were then reset with their respective baits and randomly rotated through four positions corresponding to the cardinal points. Thus, by the end of each experiment, each bait had been used once at every of the four positions, so that any site bias should not affect treatment comparisons. All baits containing eggs were transported into the lab and reared to adults in order to obtain the species identification. Air temperature was daily recorded using data loggers. Strong winds and heavy rain prevent flies from dispersing ( Digby 1958 ). Therefore, traps were only set when favorable weather was present. Collected flies were either preserved in 70% ethanol or dried. Insects were morphologically identified using identification keys ( Rognes 1991 ; Gregor et al. 2002 ; Oosterbroek 2006 ) and by specialists whenever required. Only specimens from Calliphoridae, Muscidae, Fanniidae, Anthomyiidae, and Heleomyzidae families were identified to species level. All specimens were deposited in the collection of the Department of Animal Biology in the Faculty of Sciences of the University of Lisbon.

### Statistical analysis

Absolute and relative specimen abundance and species richness were analyzed. Families and genera not identified to species level were counted as one species. To estimate the similarity of the communities captured among baits, Sørensen’s similarity coefficient (CS) was used. Its formula is: 2jN / (aN+bN), where aN is the total number of taxa in site A, bN is the total number of taxa in site B, and jN is the sum of the taxa found in both sites. This index is equal to 1 in case of total similarity and equal to zero if the sites are dissimilar and have no species in common.

Oneway ANOVA was used to test the differences among the three baits total captures. A multivariate ANOVA was performed on average values of collected specimens to analyze the effect of the two factors: type of bait and temperature. Separate ANOVA analyses were done within each forensically important species to test the effect of bait in the number of collected specimens, and a Mann-Whitney-Wilcoxon test was used to analyze the populations between the two temperature groups. The Tukey and Bonferroni tests were used to analyze all post-hoc pairwise comparison. Data were log transformed in order to get parametric tests presumptions. Differences were considered to be significant at the 0.05 level. Analyses were performed using IBM SPSS Statistics 19 (( www.ibm.com ).

### DNA extraction, amplification, and sequencing

A specimen of each Diptera identified to the species level was subject to molecular identification using the COI barcode region.

DNA was extracted from two or three legs of each specimen using the E.Z.N.A.® Insect DNA Kit (Omega Bio-Tek, ( www.omegabiotek.com ), following the manufacturer’s instructions with the exception of the elution step, which was performed in 40 µL of elution buffer in order to increase DNA concentration. COI barcode region (658 bp) was amplified using the primer pair LCO1490 (GGTCAACAAATCATAAAGATATTGG) and HCO2198 (TAAACTTCAGGGTGACCAAAAAATCA) ( Folmer et al. 1994 ). Each PCR reaction contained 1x colorless GoTaq® Reaction Buffer (Promega, ( www.promega.com ), 2 mM MgCl2, 0.1 mM dNTPs, 0.4 µM of each primer, 0.16 µg/µL of BSA, 0.25 U of GoTaq® DNA polymerase (Promega), and 4 µL of DNA template, in a final volume of 25 µL. PCR thermal conditions consisted of 1 cycle of 1 min at 94 °C; 5 cycles of 30 sec at 94 °C, 1 min at 45 °C, and 1 min at 72 °C;, 35 cycles of 1 min at 94 °C, 1.5 min at 50 °C, and 1 min at 72°C; and a final cycle of 5 min at 72°C. PCR products were verified in 1% agarose gels stained with RedSafeTM (iNtRON Biotechnology, ( www.intronbio.com ) at 100V for 25 minutes along with the molecular weight marker HyperLadderTM IV (Bioline, ( www.bioline.com ) and then purified using the commercial kit SureClean (Bioline) according to the manufacturer’s instructions. Amplicons were sequenced in both directions by the commercial facility offered by Macrogen Company (Macrogen Europe, ( www.macrogen.com ).

### DNA sequence analysis

Sequences were checked and edited in Sequencher® v4.0.5 (Gene Codes Corporation, ( www.genecodes.com ) and then they were blasted in online databases ( http://www.ncbi.nlm.nih.gov and http://www.boldsystems.org ). Sequences of all taxa identified to species level were submitted to the Barcode of Life Data Systems (BOLD Systems) database.

## Results

A total of 2371 specimens belonging to three insect orders (Diptera, Coleoptera, and Hymenoptera) were caught in four experiments between March 2011 and January 2012 ( Table 2 ). Orders Coleoptera and Hymenoptera were not analyzed. Diptera was the predominant order, with a total of 2132 adults (90.0% of all collected insects) belonging to 21 families. Calliphoridae was the dominant family of the Diptera community (39.7%), and Muscidae was the second (31.7%). Drosophilidae was the third most abundant family (21.6%), Fanniidae the fourth (7.1%), Sarcophagidae and Phoridae were the fifth and sixth, with the same abundance (6.3% each), and Anthomyiidae was the seventh (5.1%). Fifteen families (mainly non-Calyptrate) were uncommon in the traps (< 2%) ( Table 2 ).

**Table 2.**
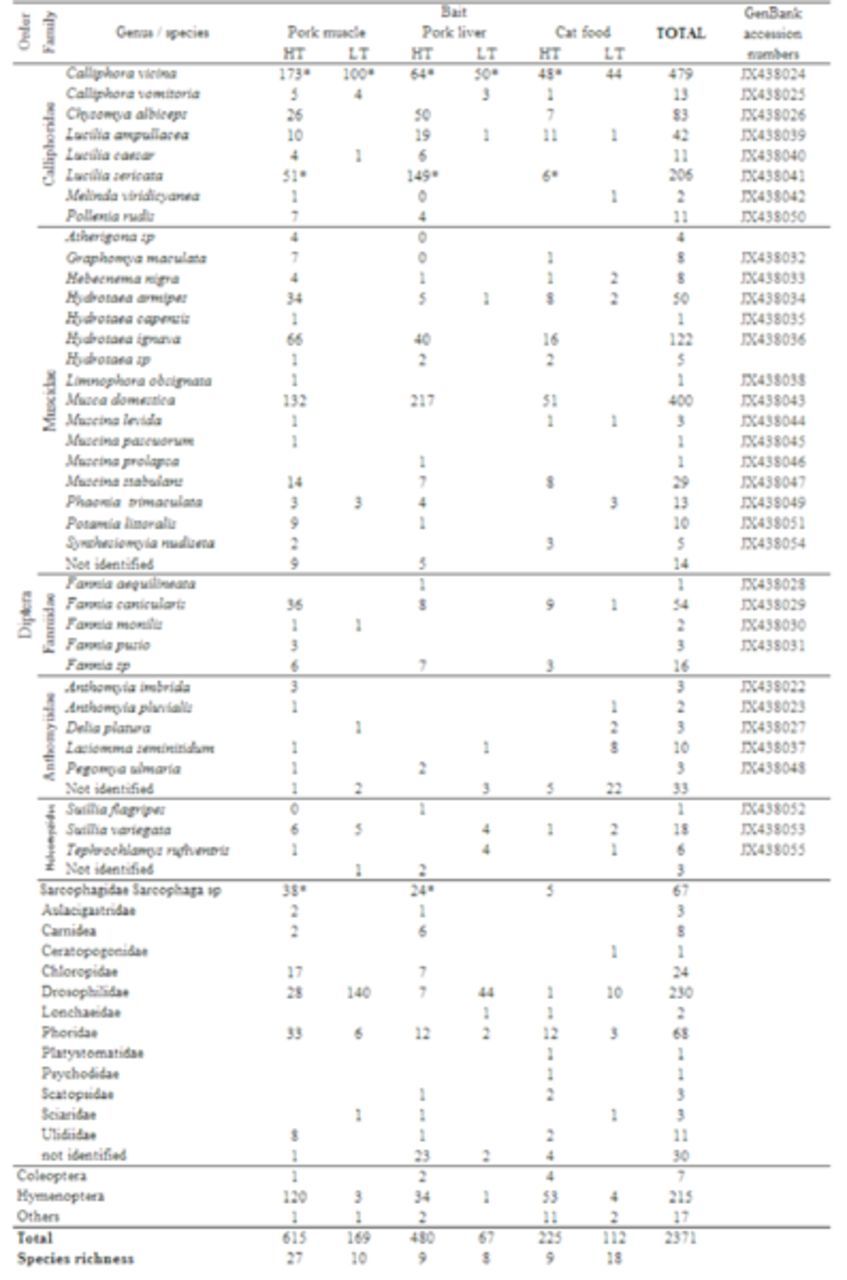
Insects collected in Lisbon, Portugal, between March 20 II and January 2012, sorted by abundance and bait attraction in each season.

*Oviposition; HT: high temperatures; LT: low temperatures.

**Table 3.**
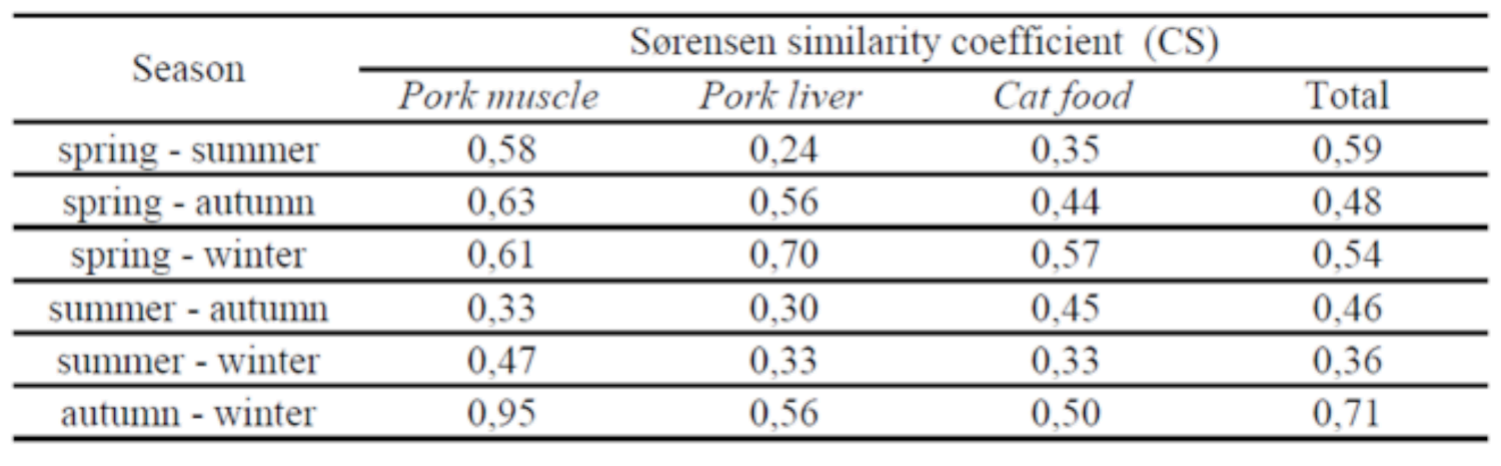
Similarity between different seasons and in each bait, expressed by the quantitative Sørensen similarity coefficient (CS).

**Table 4.**
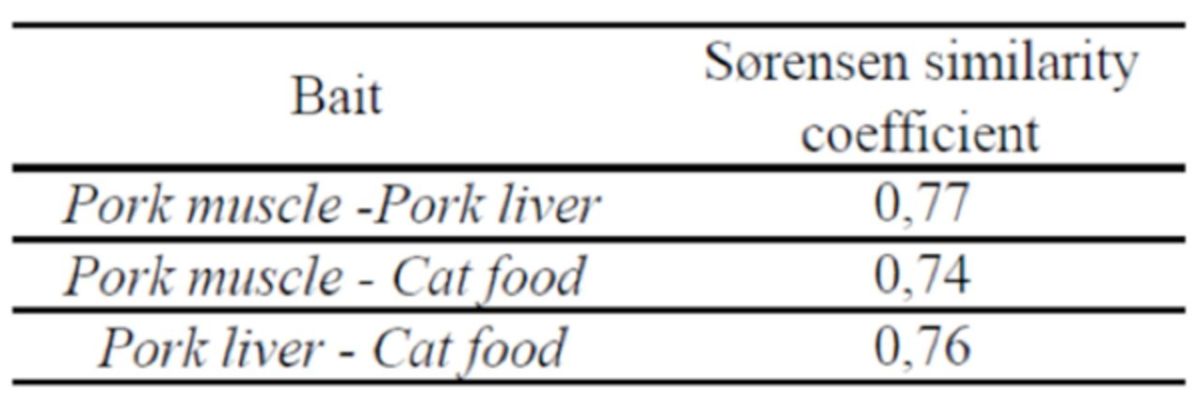
Similarity between the different baits, expressed by the quantitative Sørensen similarity coefficient (CS).

### Bait variability

From the 2371 collected specimens, 1146 were gathered in pork muscle traps (48.3 %), 834 in pork liver traps (35.2 %) and 391 in cat food traps (16.5 %). An ANOVA test revealed that there are statistical differences between the three baits captures (F = 8.597, *P* < 0.01). Posteriori Tukey and Bonferroni comparisons showed that pork muscle was significantly different from both pork liver ( *P* < 0.05) and cat food ( *P* < 0.01), the last two being not significantly different from each other.

Regarding species richness, pork muscle was the bait with the highest number of species trapped, followed by pork liver bait and finally cat food ( Table 2 ). However, when the collected populations of each bait were analyzed two by two using the Sørensen’s similarity coefficient, few differences were found. All comparisons presented CS values ranging between 0.74 (for pork muscle and cat food comparison) and 0.77 (for pork muscle and pork liver). The comparison between pork liver and cat food presented a CS of 0.76 (Table 3).

*Calliphora vicina* Robineau-Desvoidy was the most abundant species (22.0% of all Diptera captured), followed by *Musca domestica* L., and *Lucilia sericata* (Meigen) ( Table 2 ).

Concerning bait preferences, *C. vicina* was the dominant species in pork muscle and cat food baits, but with no significant preference for either of the three baits. *L. sericata* presented a statistically significant preference ( *P* < 0.05) to pork liver bait.

*Chrysomya albiceps* (Wiedemann), *Hydrotaea ignava* (Harris), and the genera *Fannia* Robineau-Desvoidy and *Sarcophaga* Meigen presented no significant preference for any of the baits, although all had very low capture numbers in cat food bait.

**Figure 1.**
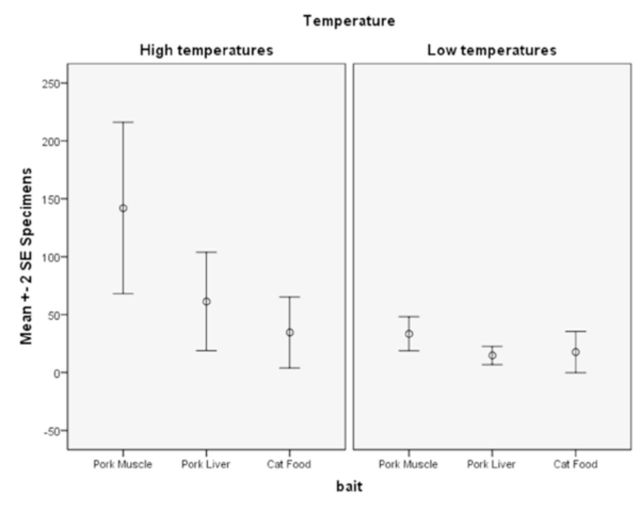
Distribution of collected specimens by bait in each temperature group. High quality figures are available online

Concerning the variable temperature, significant differences in mean captures among the three baits in each temperature group were found (high temperature: F = 4.440, *P* < 0.01; low temperature: F = 3.680, *P* < 0.05, respectively), as well as in each bait between the two temperatures ( *P* < 0.01), as warmer temperatures attracted more insects, representing 79.0% of the total number of specimens captured ( Figure 1 ).

In terms of species richness, warmer temperatures provided higher values ( Table 2 ). Taxa similarity of captured populations for each bait between the two temperature groups revealed a very low Sørensen’s similarity coefficient, being below 0.40 for all baits (Table 4).

Of the forensically important flies collected, *C. vicina* was the dominant species in average low temperatures. *L. sericata* , *L. caesar, L. ampullacea* , *C. albiceps* , *H. ignava* , *M. domestica* , and the genera *Fannia* and *Sarcophaga,* all showed high numbers of captures when average air temperature was above 16ºC. Moreover, except for *Fannia* sp., none of these species was even collected in the experiments with average temperatures below 16ºC. All these taxa showed significant abundance differences between the high and low average temperature groups ( *P* < 0.01).

Regarding oviposition in warmer temperatures, it occurred in all three baits, with both *L. sericata* and *Sarcophaga* sp. laying eggs. In the lower temperature experiments, oviposition was only observed in two baits, pork muscle and pork liver, and *C. vicina* was the only species that oviposited.

When both bait and temperature were analyzed together, no interaction was found between the two variables, i.e., the effects of both variables are independent (F = 0.038; *P* > 0.05).

### Small bait trap efficiency: comparison with other studies

The results obtained in the present study regarding the presence of forensically important Calliphoridae and Muscidae species were compared with those reported in literature from the Iberian Peninsula and are discussed below in the Discussion section ( Tables 5 , 6 ).

**Table 5.**
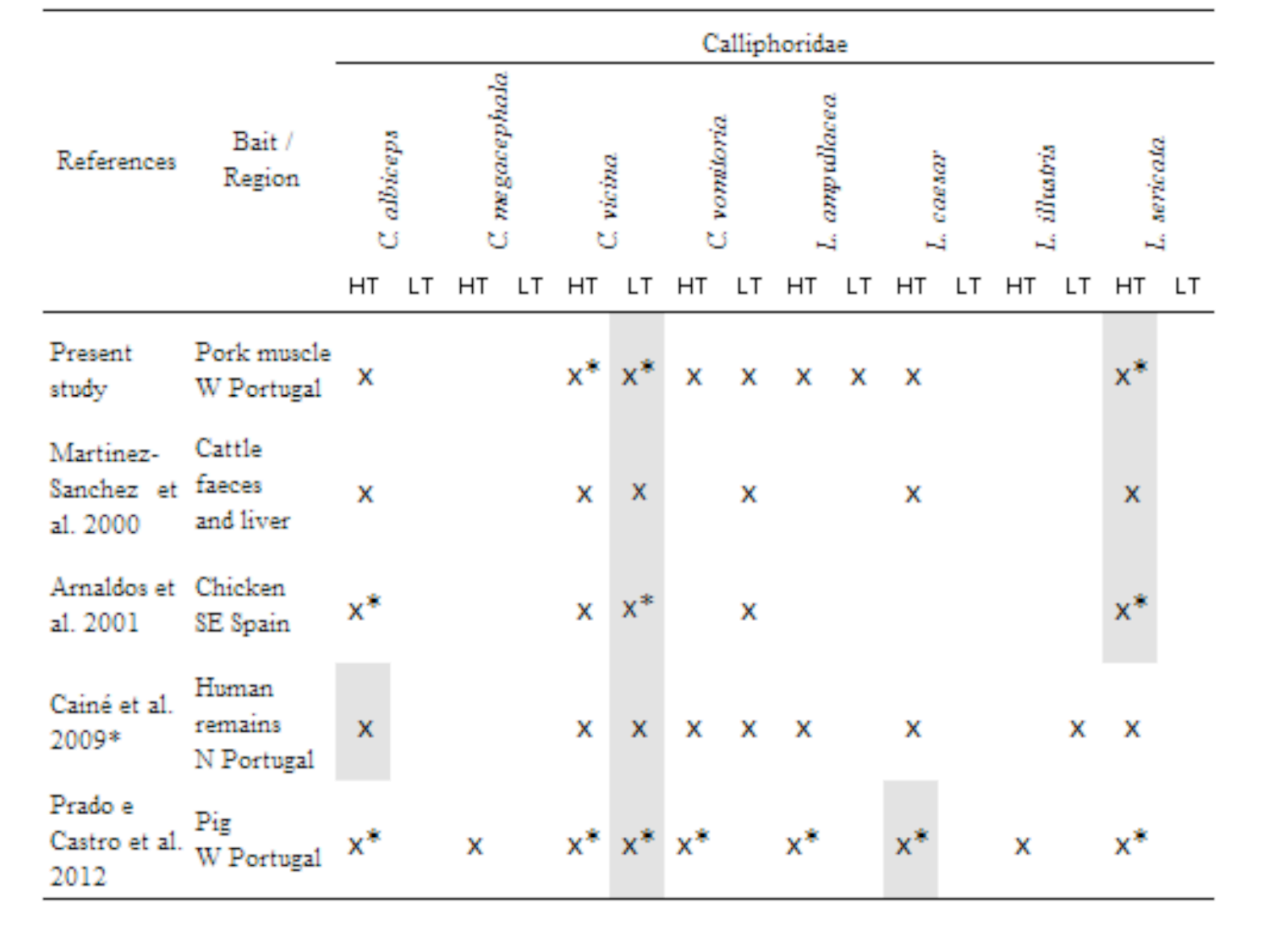
Species checklist of Calliphoridae flies from four Iberian studies and those collected in the current study.

*Oviposition; HT: high temperatures; LT: low temperatures. Shaded squares represent the predominant species.

**Table 6.**
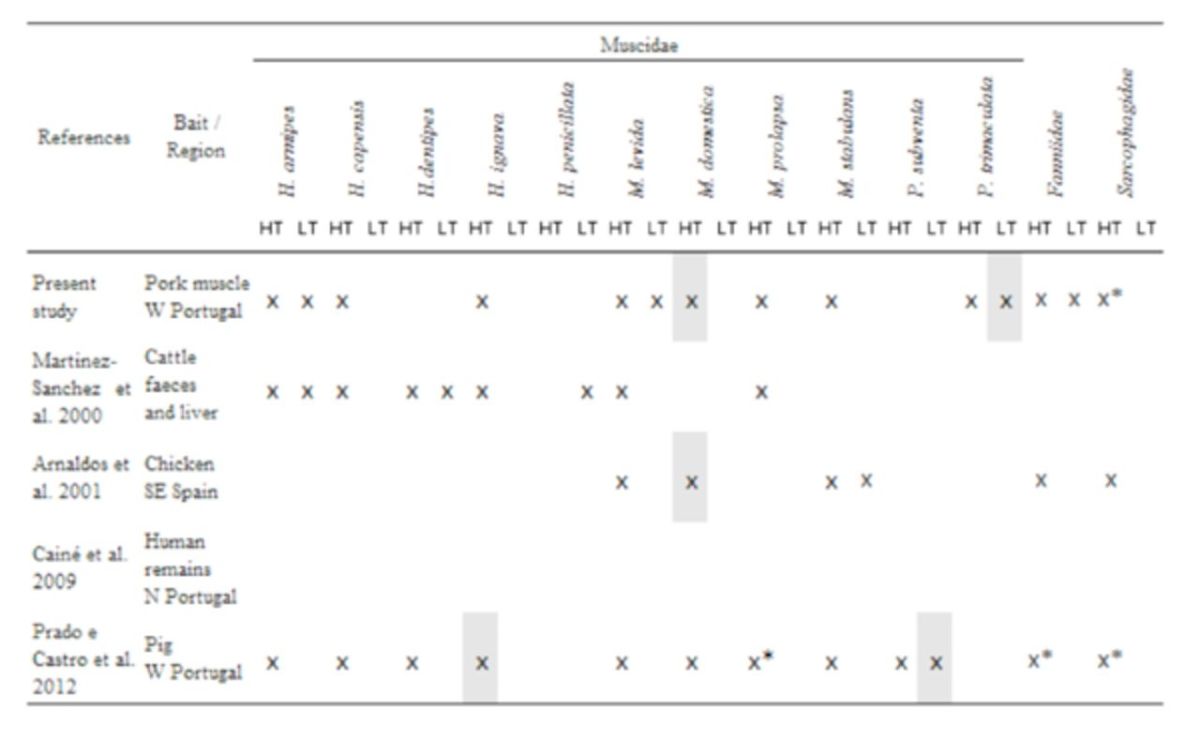
Species checklist of Muscidae and families Fanniidae and Sarcophagidae flies from four Iberian studies and those collected in the current study.

*Oviposition; HT: high temperatures; LT: low temperatures. Shaded squares represent the predominant species.

### Molecular identification through COI barcode region

From each one of the 34 species identified based on morphologic criteria ( Table 2 ), a specimen was identified at the molecular level and each COI barcode sequence was submitted to BOLD Systems online database. A successful identification is achieved when the nucleotide sequence of the specimen matches to the available reference sequences, leading to a species match. Nevertheless, online databases are still growing and many species do not have a reference sequence to be compared with. Of the 34 species morphologically identified, only 15 were correctly identified at the species level in BOLD database: *Calliphora vicina* , *Calliphora vomitoria* (L.), *Chrysomia albiceps* , *Lucilia ampullacea* (Villeneuve), *Lucilia caesar* (L.), *Lucilia sericata* , *Graphomyia maculata* (Scopoli), *Musca domestica* (L.), *Muscina levida* (Harris), *Muscina stabulans* (Fallen), *Potamia littoralis* (Robineau-Desvoidy), *Synthesiomyia nudiseta* (van der Wulp), *Fannia canicularis* (L.), *Anthomyia pluvialis* (L.), *Delia platura* (Meigen). Nine species were identified at the genus level, *Melinda viridicyanea* (RobineauDesvoidy), *Pollenia rudis* (Fabricius), *Hydrotaea armipes* (Fallen), *Muscina pascuorum* (Meigen), *Muscina prolapsa* (Harris), *Fannia aequilineata* (Ringdahl), *Fannia monilis* (Haliday), *Anthomyia imbrida* (Rondani), and *Pegomya ulmaria* (Rondani), six to the family level, *Hebecnema nigra* (RobineauDesvoidy), *Hydrotaea capensis* (Wiedemann), *Hydrotaea ignava* , *Limnophora obsignata* (Rondani), *Fannia pusio* (Wiedemann), and *Lasiomma seminitidum* (Zetterstedt), and four only to order level, *Phaonia trimaculata* (Bouché), *Suillia flagripes* (Czerny), *Suillia variegata* (Loew), and *Tephrochlamys rufiventris* (Meigen).

## Discussion

### Species composition and relative abundance

As in former forensic studies carried out in the Iberian Peninsula, the community of sampled Diptera was dominated by the families Calliphoridae and Muscidae ( Martínez-Sánchez et al. 2000 ; Arnaldos et al. 2004 ; Baz et al. 2007 ; Prado e Castro et al. 2012 ). However, in contrast with these authors, this study presents Drosophilidae as the third most abundant family. This can be due to the type of bait or the type of trap used (totally covered in compact plastic instead of wire mesh), which did not allow any fly, even the smallest, to escape. Despite having already recovered some larvae of this family in human autopsies performed in Madrid, its forensic importance is still limited due to lack of knowledge of its biology and distribution in the Iberian Peninsula (Velásquez et al. 2010). Thus, it was not evaluated in the present study.

### Bait variability

Analyzing the factor bait, it was the pork muscle that captured the highest number of specimens and species. This result might be due to the fact that this bait had an outer layer that was not as dry as the pork liver and cat food during the experiments, which kept the odor stronger (personal observation). This feature might also be the explanation for its large masses of eggs, as ecological studies carried out by Barton-Browne (1962) showed that female carrion breeding sites depend on tarsal contact with water or moisture. In fact, some studies aiming to estimate primary forensic colonizers also use pork muscle as bait (e.g., Oliva 2001 ; Leccese 2004 ; Baz et al. 2007 ). In all experiments, the cat food was the least attractive. The absence of eggs in this bait in cold temperatures, when there are fewer active insects, leads to the assumption that it is less preferable to carrion flies and, consequently, not a good substrate to estimate primary Diptera colonizers.

When forensically important taxa were analyzed individually, only *L. sericata* presented significant differences of abundance among baits, showing a possible preference for pork liver.

### Temperature effects on species abundance and richness

The occurrence of carrion flies was clearly influenced by temperature effects, as expected from the literature ( Tantawi et al. 1996 ; Martínez-Sánchez et al. 2000; Schrӧeder et al. 2003 ; Arnaldos et al. 2004 ; Grassberger and Frank 2004 ; Hwang and Turner 2005 ; Anton et al. 2011 ; Battán Horenstein and Salvo 2012 ; Prado e Castro et al. 2012 ; George et al. 2013 ). The highest number of specimens and species was caught with a high average air temperature, while the lowest values were obtained in low temperatures, which is in agreement with the results obtained from western Spain by Martínez-Sánchez et al. (2000) . However, Prado e Castro et al. (2012) , in a study conducted in Lisbon, as was the present one, using pig carcasses as bait, presented higher values of specimen abundance in spring, a mild season. This inconsistency may be due to differences in size and type of bait, differences in specific biotopes, or temperature differences between sampling years, as Prado e Castro conducted the study in 2006, when spring had an average temperature of 18.9ºC, which was abnormally above the mild seasons average. These climate changes from year to year within seasons highlight the importance of the variable air temperature instead of calendar limitations ( Turchetto and Vanin 2004 ). However, it is important to note other variables as well, such as the circadian cycle and air humidity ( Nuorteva 1959 ; Anderson 2001; Campobasso et al 2001 ; George et al. 2013 ).

Given the abundance of the dominant species at each temperature, we can classify them into groups, the apparent thermophobic ( *C. vicina* ) and the apparent thermophilic ( *L. sericata* , *L. caesar* , *C. albiceps* , *M. domestica* , *H. ignava* , *Fannia* sp., and *Sarcophaga* sp.), all with abundance peaks in the warmer temperatures.

### Small bait traps as an efficient alternative to capture first colonizers: comparison among studies

The overall population of early colonizers collected in each temperature experiment regarding pork muscle bait, because of its higher species richness and abundance results, was compared with populations obtained, for the first seven days, in forensic studies using animals carcasses conducted in the Iberian Peninsula ( Tables 5 , 6 ). The aim was to determine whether small bait traps are a viable alternative to other traps with ethical and practicability constraints.

Records from studies conducted in Salamanca, Spain ( Martínez-Sánchez et al. 2000 ), less than 500 km away from our sampling sites, showed a similar taxonomic composition in regard to blowfly species, with all but one species ( *L. ampullacea* ) collected here not being recovered at Salamanca study sites ( Table 5 ). A study carried out in Lisbon ( Prado e Castro et al. 2012 ) presented some differences with the present one in the high temperature, namely, the dominance of *L. caesar* instead of *L. sericata* concerning family Calliphoridae and the dominance of *H. ignava* in summer in place of *M. domestica* regarding family Muscidae. These differences are consistent with the rural environment in the sampling area of Prado e Castro et al. (2012) and the more urban environment of the present study ( Anderson and Vanlaerhoven 1996 ; Schrӧeder et al 2003 ; Hwang and Turner 2005 ; Brundage et al 2011 ). Nevertheless, all blowfly species, except for *Chrysomya megacephala* , which was collected on pig carcass in Lisbon ( Prado e Castro et al. 2012 ) and also on chicken carcass in Murcia, Southeast Spain ( Arnaldos et al. 2004 ), were also collected in the present study. It is important to highlight that *C. megacephala* is a species only recently discovered in Portugal and in low numbers ( Prado e Castro and García 2009 ). Conversely, most of the Diptera species from family Muscidae documented on the first seven days in carcasses in Salamanca ( Martínez-Sánchez et al. 2000 ) were not part of the insect fauna of our study. Although, the study performed in Murcia (Arnaldos et al. 2004) and the one performed in Lisbon ( Prado e Castro et al. 2012 ) found the same species of muscid flies as the ones found in this study, except for the species *H. dentipes* was collected in Prado e Castro et al. 2012 but not here, and a trade in the genera *Phaonia* , as the present study collected the species *Phaonia trimaculata* and Prado e Castro et al. collected *P. subventa* ( Table 6 ). These results suggest that forensically important Muscidae communities are more region-specific and/or more bait sensitive than Calliphoridae. All species but *Lucilia illustris* documented in human autopsies in northern Portugal ( Cainé et al. 2009 ) were also collected in this study. Nevertheless, caution should be taken respecting the difficulty in the morphologic and molecular differentiation of *L. caesar* and *L. illustris* , which can lead to misidentifications ( Oliveira et al. 2011 ; Sonet et al. 2012 ).

This analysis suggests that bottle traps with pork muscle as bait are able to capture important early colonizers of carrion and thus can be accurate predictors of the Diptera primary carrion community, in terms of species presence, in a given area. In fact, the traps used in this study, as well as the experimental design, captured the same diversity of Diptera early colonizers when compared with studies using pig carcasses in the same region ( Prado e Castro et al. 2012 ). Moreover, this kind of trap is cheap, easy to make and replicate, and does not have the ethical, legal, or practicability implications associated with the use of human remains or pig carcasses. Gruner et al. (2007) had already found that aerial capture of adult specimens accurately predicted carrioncolonizing species. With respect to bait, other authors using meat baits or small carcasses have already shown that these substrates are suitable for the development cycles ( Leccese 2004 ) and population estimation of the early necrophagous Diptera species ( Leccese 2004 ; Arnaldos et al. 2005 ; Brundage et al. 2011 ) even though the size of these baits is not comparable to a human body. In fact, the odors emitted from meat baits should contain many of the volatile organic compounds emitted from an entire freshly dead mammal, such as pigs, the most common bait used in forensic studies ( George et al 2013 ).

### Molecular identification

In this study, identification of forensically important Diptera was conducted both at a morphological and molecular level. Molecular identification has become an indispensable tool in forensic scenarios. The DNA barcoding project (( www.barcodeoflife.org ) aims to assign every species with a unique sequence (barcode sequence), allowing a reliable and error free identification of a specimen. However, this method has its constraints. Closely related species may exhibit a very similar nucleotide sequence, and this can lead to a misleading species determination. Examples of this are *L. caesar* and *L. illustris* , which are not distinguished through COI barcode region ( Wells et al. 2007 ; Oliveira et al. 2011 ). Also, the lack of reference sequences for a great number of species, mainly due to a poor knowledge of species catalogue in some regions, leads to identifications only to genus, family, or even order level. This last constraint was present in this study, as less than 50% of the specimens, previously identified morphologically, were blasted in BOLD Systems database at species level. Few molecular studies on forensically important insects using COI barcode region have been done in Portugal, except for the most abundant forensic Diptera ( Cainé et al. 2006 , 2009; Ferreira et al. 2011 ; Oliveira et al. 2011 ; Rolo et al. 2013 ). All sequences were added to the BOLD database and are an important contribution to future species determination through molecular methodologies.

### Conclusions

This study was the first one carried out in Portugal comparing small-bait efficiency in estimating populations of first colonizers in a specific region. Molecular identification of several Diptera species through COI barcode region, including several species in which such approach was performed for the first time, contributes to increase online databases, thereby expanding the reliability and unequivocal species determination, which is essential in many scientific fields, namely in general entomology and in medical-legal investigations.

Although these results have limited applicability in criminal investigations of bodies discovered after a long period of time (more than one week), the method described here proved to be efficient and prompt in estimating the population of early Diptera colonizers. Therefore, it is a reliable tool to acquire useful knowledge to be applied in investigations on fresh body findings, as it easily records the primary Diptera sarcosaprophagous fauna of the area where the body was discovered. These inventories can help criminal investigations in the identification of the species collected from the cadaver, human or animal, in indicating correct times of oviposition, and also signaling the original location of a crime.

## Abbreviations

CS: Sørensen similarity coefficient
COI: cytochrome *c* oxidase I
